# The Mansion House and the Hospitals

**Published:** 1902-10-25

**Authors:** 


### THE MANSION HOUSE AND THE HOSPITALS.

The Mansion House has for long been rightly regarded by the English people as the centre from which most great benevolent efforts emanate. We showed a few years ago that, during the last 25 years, the influence and authority of the Mansion House in matters of charity has increased and extended for good among all classes from the highest to the lowest. In the quarter of a century ending with the close of 1900, upwards of four and three-quarter millions sterling were raised for charitable purposes by the agency of the Mansion House funds. As a channel for raising money the Mansion House has an unblemished record, and has never failed to successfully carry through every effort which has been started under its auspices during the period in question. Everyone is justly confident, in sending a cheque to the Mansion House, that the funds entrusted to the Lord Mayor for the time being will be economically administered upon business principles, so that the full value of each gift may be expended upon the object which the giver desires to further. During the last 27 years a sum approaching three millions sterling has been raised for the hospitals through the Mansion House. It might be thought that people would tire of sending money to the same place for various objects year by year. This is, however, not the case, for the contributions to charity through the Mansion House have increased by leaps and bounds, so that, whereas in 1876 the total contributions paid into the Mansion House amounted to but £73,000, during 1900 upwards of £1,650,000 was contributed to charity through this one source alone.

In the Coronation year the great personal interest which the King is known to take in hospitals, and the fact that the Lord Mayor shared this feeling, have contributed to secure a result which will make Sir Joseph Dimsdale's mayoralty memorable. On the whole the Mansion House in the Coronation year has done so much to help the medical charities as to constitute a record in well-doing for the hospitals. The sum raised for the Coronation gift amounts to £117,000. Many hospitals, notably Guy's and the London fever hospitals, have largely benefited. But the most remarkable, and at the same time the most gratifying, fact is that the contributions on Hospital Sunday—upwards of £63,000—constitute a record as to amount, and, what is even more important, they mark a new departure, and afford evidence of a volume of new life infused by the action of the Lord Mayor into the contributing congregations throughout the metropolis. The result of the scheme which the Lord Mayor himself proposed, coupled with the cordial and hearty co-operation of the clergy and ministers of all denominations, have increased the sum received collectively from the churches during 1902 to £46,000, a sum which is £10,000 more than the average of the collections for many years past. There can be no doubt that the new life thus given to the Hospital Sunday Fund will extend and develop, and that there is now every prospect that within a measurable time the total contributions from this source will be raised to £100,000 a year, an amount which London is quite capable of giving without difficulty through the earnest co-operation of the churches. We feel too that the action of the Lord Mayor has stirred up the press as well as the churches, and that the combination of press and pulpit in the Coronation year has produced such important results, that this unanimity in action and purpose may be calculated to continue and extend in the future. We wish Sir Joseph Dimsdale and the Lady Mayoress continuous happiness, for to them the London hospitals owe great benefit, and we can hope and believe that they will both look back with the utmost satisfaction to the good work which they have been able to accomplish in the cause of suffering and the sick.

It is only just, when dealing with this subject, to bear testimony to the admirable manner and untiring work of Sir William Soulsby, C.B., who has been private secretary to successive Lord Mayors for twenty-seven years. His mastery of detail, his accurate knowledge of individuals and institutions, his tact, resource and agreeable manner, make Sir William Soulsby one of the forces of our modern life in the metropolis of the Empire. We beg to express to Sir William Soulsby on behalf of the whole of the institutions of every class, and of the managers generally, the most grateful acknowledgments for his self-denying efforts and that of his able assistants, who have deservedly won the full confidence of the generous of all classes, and are widely recognised as deserving of the highest esteem and honour.

